# Treatment of noninfectious uveitis

**DOI:** 10.5935/0004-2749.2022-0094

**Published:** 2025-08-21

**Authors:** Lisia Barros Ferreira, Alexandra L. Farrall, João M. Furtado, Justine R. Smith

**Affiliations:** 1 College of Medicine & Public Health, Flinders University, Adelaide, Australia; 2 Division of Ophthalmology, Faculdade de Medicina de Ribeirão Preto, Universidade de São Paulo, Ribeirão Preto, SP, Brazil

**Keywords:** Uveitis, Noninfectious uveitis, Immunomodulatory therapy, Biologics, Inflammation, Uveítes, uveíte não-infecciosa, Terapia imunomodulatória, Biológicos, Inflamação

## Abstract

Uveitis is a broad term that refers to a large group of eye disorders categorized by intraocular inflammation, a leading cause of visual impairment. Historically, treatment of noninfectious uveitis has depended on corticosteroid drugs. Owing to the myriad of side effects caused by corticosteroids, immunomodulatory therapy has become the preferred treatment for chronic noninfectious intraocular inflammation. Recently, biological response modifiers have established a new era in uveitis therapy, with the range of targets continuing to expand. In this review, we aimed to convey up-to-date information on the treatment of noninfectious uveitis to the general ophthalmologist.

## Abstract (pt)

Uveíte é um termo amplo utilizado para denominar várias desordens categorizadas como inflamação intraocular, uma causa importante de deficiência visual. Historicamente, o tratamento das uveítes não infecciosas baseou-se no uso de corticosteróides. Devido aos diversos efeitos colaterais do uso de corticosteróides a longo prazo, a terapia imunomoduladora é indicada no tratamento das uveítes não infecciosas crônicas. A introdução dos medicamentos biológicos estabeleceu uma nova era no tratamento das uveítes, com constante desenvolvimento de novas drogas. O objetivo desta revisão é trazer informações atuais sobre tratamento das uveítes não infecciosas para a prática clínica do oftalmologista geral.

## INTRODUCTION

Uveitis is one of the leading causes of visual impairment in the Western world^(1)^. The goals of uveitis therapy are to suppress inflammation, achieve inactivity of the disease, and prevent secondary complications that may lead to vision loss. An interdisciplinary clinical team is often required to achieve the most comprehensive and effective care. Corticosteroids are the cornerstone of noninfectious uveitis care^(2)^. Topical, regional, and systemic forms are used, depending on the underlying etiology and clinical presentation of uveitis. When local corticosteroid treatment is insufficient or inappropriate to control intraocular inflammation, systemic corticosteroid therapy is prescribed, but the dose must be tapered as soon as possible owing to the extensive list of possible adverse side effects arising from long-term use. Immunomodulatory therapy should be implemented if prolonged use of corticosteroids is necessary to suppress the inflammation, usually starting with conventional immunomodulatory drugs and adding, or switching to, biologic response modifiers if required^(3)^. Herein, we review the drugs currently used for care of noninfectious uveitis, including delivery and dosing considerations, and side effects.

## LOCAL TREATMENT

### Topical treatment

#### Topical corticosteroids

Corticosteroids at the top of the inflammatory cascade, downregulating many inflammatory mediators, partly via inhibition of the phospholipase A2 and cyclooxygenase-2 activities^(4)^. Corticosteroid eyedrops are used primarily for anterior uveitis. The administration frequency is defined by the grade of anterior chamber inflammation and may range from every hour to once every other day, usually initiated intensively, with slow tapering over weeks to months^(3)^. The commonly used drugs are dexamethasone and prednisolone acetate, the latter reaching higher concentrations in the aqueous humor^(5)^. Difluprednate has been demonstrated to reach the posterior segment at significant concentrations and is effective in the treatment of cystoid macular edema^(6,7)^. Difluprednate causes higher elevations of intraocular pressure^(7,8)^, whereas less potent corticosteroids, such as fluorometholone and loteprednol, have less impact on intraocular pressure but are also not as effective in tackling inflammation^(9)^. Other common side effects of topical corticosteroids include cataract and glaucoma. Table 1 presents the most commonly used topical corticosteroids.

**Table 1:** Topical corticosteroids and their side effects

Drug	Side effects
Prednisolone acetate 1%	Ocular hypertension, glaucoma, cataract, ocular surface infections, and eyelid skin atrophy
Prednisolone sodium phosphate 1%	
Dexamethasone sodium phosphate suspension 0.1% and ointment 0.05%	
Difluprednate 0.05%	
Fluorometholone suspension 0.1% and 0.25% and ointment 0.1%	

#### Topical non-steroidal anti-inflammatory drugs

Topical non-steroidal anti-inflammatory drugs (NSAIDs), which suppress the cyclooxygenase-1 and 2 enzymes, are not used for managing inflammation in uveitis but may play a role in the treatment of cystoid macular edema secondary to uveitis^(10)^. Although rare, corneal melting and perforation have been described as adverse reactions^(11)^.

#### Topical cycloplegics

Tropicamide, cyclopentolate, and atropine are cycloplegic drops prescribed to reduce ciliary spasm and the associated ocular pain and photophobia, and to prevent the development of posterior synechiae in the setting of anterior uveitis^(3)^. Cyclopentolate should be used cautiously in young children under 6 years because of the risk of central nervous system disturbances^(12)^. The duration of cycloplegia for each drug is described in table 2. Tropicamide has a less potent and shorter cycloplegic effect, making it a popular choice^(13)^.

**Table 2:** Cycloplegic agents, duration of cycloplegia, dosing, and side effects

Drug	Duration of cycloplegia	Dosing	Side effects
Tropicamide 1%	2-6 hours	2-4 times daily	Conjunctival hyperemia, photophobia, blurred vision, and neurologic side effects (in children)
Cyclopentolate 0.5% and 1%	12-24 hours	2-4 times daily	
Atropine 0.5% and 1%	7-14 days	1-2 times daily	

### Regional treatment

Regional corticosteroid treatment is administered as periocular or intraocular injections, or intraocular implants to treat the posterior segment of the eye. This has the advantage of not exposing the patient to systemic complications and is commonly used for unilateral uveitis. Table 3 shows injected and implanted corticosteroids and their associated side effects.

**Table 3:** Periocular and intraocular corticosteroid compounds and side effects

Administration	Compound	Side effects
Periocular - posterior subtenon	Triamcinolone acetonide 40 mg/mL	Ptosis, periocular hemorrhage, chemosis,
Periocular - orbital floor	Triamcinolone acetonide 40 mg/mL	globe perforation, and orbital fat herniation
Intraocular	Triamcinolone acetonide 2-4 mg/0.05-0.1 mL	Ocular hypertension, cataract, retinal
Intravitreal implant	Fluocinolone 0.59 mg (Retisert) Fluocinolone 0.19 mg (Iluvien) Dexamethasone 0.7 mg (Ozurdex)	detachment, vitreous hemorrhage, and endophthalmitis

#### Periocular corticosteroids

Periocular corticosteroid injections include subtenon and orbital floor approaches. In the posterior subtenon approach, a superotemporal injection of triamcinolone acetonide is given, with effects usually lasting for 3-4 months^(14)^. Periocular corticosteroids may take 2 to 6 weeks to achieve the maximum therapeutic effect and so are not the optimal choice when control of inflammation is urgent^(15)^. Eyelid ptosis, periocular hemorrhage, and globe perforation are the main reported risks^(16)^. Orbital floor injections may induce orbital fat herniation^(17)^. Posterior subtenon corticosteroid injections are effective in treating uveitic macular edema in >50% of patients within 3 months^(14)^. An intraocular pressure of ≥24 mmHg has been reported in approximately half of patients, with approximately 2% and 14% requiring glaucoma and cataract surgery, respectively, within 12 months^(15)^. Subconjunctival dexamethasone attains high concentrations in aqueous humor but unsatisfactory diffusion to the vitreous^(18)^.

#### Intravitreal corticosteroids

Intravitreal injections of corticosteroids are preferred to obtain a rapid anti-inflammatory effect and treat macular edema refractory to periocular corticosteroids. Triamcinolone acetonide is administered via pars plana injection with a 27-gauge needle, with therapeutic effects lasting for 3-6 months in non-vitrectomized eyes^(19)^. The intraocular pressure may start to increase 1 week after the intravitreal injection procedure and may remain elevated for up to 9 months, with children being more prone^(20)^. Up to 100% of eyes treated with intravitreal triamcinolone need topical or systemic anti-glaucoma therapy, and small numbers will require filtering surgery^(20,21)^. Posterior subcapsular cataract may develop and progress in 50% of cases^(22)^. Sterile endophthalmitis is reported in up to 4.6% of cases of intravitreal triamcinolone injection, mostly when preserved and small particle formulations are used^(23)^. As with any other intraocular procedure, intravitreal injection also carries a risk of infectious endophthalmitis.

Corticosteroid implants were developed to last for long durations and to achieve effective concentrations of drugs inside the eye. Retisert (Bausch and Lomb, Inc.), the first implantable corticosteroid released in 2005 for the treatment of chronic noninfectious uveitis, is composed of 0.59 mg of fluocinolone acetonide and implanted via a small pars plana incision, with a therapeutic effect lasting up to 30 months^(24)^. Within 24 months, a substantial proportion of eyes require pressure-lowering medications (60%) or filtering surgery (25%). Cataract surgery is required in 80% of eyes^(24)^. Displaying a safer profile, Ozurdex^®^ (Allergan) is an implant containing 0.7 mg of dexamethasone administered via pars plana injection with a 22-gauge needle applicator, and its effects last up to 6 months^(25)^. During this time, 7% of eyes have increased intraocular pressure (≥25 mmHg) and 15% develop cataract^(25)^. Corticosteroid implants should be avoided in patients with aphakia and/or a displaced intraocular lens as a result of the risk of implant migration to the anterior chamber. Intravitreal dexamethasone implant or intravitreal triamcinolone injection are superior to posterior subtenon triamcinolone injection for the treatment of uveitic macular edema^(26)^.

Approved for diabetic macular edema treatment in 2014, the Iluvien implant (pSivida Corp; 0.19 mg fluocinolone acetonide), inserted using a 25-gauge needle applicator, may provide 36 months of sustained release of fluocinolone. Increased intraocular pressure and cataract are reported in 37% and 43% of patients, respectively^(27)^. Yutiq (Eyepoint Pharmaceuticals) is a microinsert containing 0.18 mg of fluocinolone acetonide, which is also available for the treatment of noninfectious posterior segment intraocular inflammation^(28)^.

#### Other intravitreal drugs

Several drugs that are usually administered systemically (see below) have been also delivered intravitreally. Intravitreal methotrexate (400 µg/0.1 mL) injections, a treatment initially developed for ocular involvement in primary central nervous system lymphoma^(29,30)^, is an option that may effectively control intraocular inflammation and induce long periods of remission^(31)^. Intravitreal sirolimus (440 µg/0.02 mL) has been studied for the treatment of noninfectious intermediate and posterior uveitis and was shown to control inflammation in around 50% of patients^(32)^.

## SYSTEMIC TREATMENT

### Systemic corticosteroids

Systemic corticosteroids are effective and widely used despite their broad range of side effects. When topical or regional treatments are insufficient or not appropriate to control intraocular inflammation, systemic corticosteroid therapy is initiated. Depending on the severity of the clinical presentation, oral prednisone at an initial dose of 1 mg/kg/day, with tapering over approximately 3 months, should be used in an individualized manner^(3)^. Pulse therapy with intravenous methylprednisolone (1 g/ day for 3 days) may be used for severe sight-threatening cases such as the acute presentation of Vogt-Koyanagi- Harada (VKH) syndrome^(33)^. This intravenous therapy is followed by a course of oral prednisone therapy.

Complications include osteoporosis, diabetes mellitus, systemic hypertension, weight gain, mood changes, peptic ulcer, pancreatitis, hirsutism, femoral head osteonecrosis, infections, cataracts, and glaucoma^(34)^. Growth retardation is a specific concern in children who receive long-term treatment. Patients taking chronic doses of corticosteroids should take vitamin D and calcium supplements to mitigate bone density loss, and may require histamine-2 receptor blockers or proton pump inhibitors for gastroesophageal protection.

### Systemic non-steroidal anti-inflammatory drugs

Systemic NSAIDs are not generally used in the treatment of uveitis. However, a role has been suggested in the prevention of ocular inflammation, particularly when used in combination with tumor necrosis factor

(TNF)-α blocking drugs in patients with ankylosing spondylitis^(35)^.

### Immunomodulatory therapy

When an oral prednisone dose >5 mg/day is required beyond 3 months or when inflammation persists despite corticosteroid use or corticosteroid use is not tolerable, immunomodulatory therapy is the next treatment approach^(36)^. Immunomodulators modify the immune response through different pathways in conjunction with the corticosteroid-sparing effect. As it may take several weeks to months for immunomodulatory therapy to control inflammation, concomitant corticosteroid use is advised until the full effect is achieved.

Immunomodulatory therapy is divided into conventional and biologic response modifier drugs. Biologic response modifiers, or “biologics”, are a class of immunomodulatory drugs that target different inflammatory molecules such as interleukins (IL) and TNF-α. Biologic response modifiers are usually second-line to the conventional immunomodulatory drugs for the treatment of recalcitrant cases of noninfectious uveitis.

A thorough clinical history taking and laboratory assessment should be performed before initiating immunomodulatory treatment, with close monitoring during follow-up^(36)^. Baseline blood cell counts, and liver and renal functions must be assessed, along with evaluation to rule out active or latent infectious diseases such as tuberculosis. Personal and familial histories of cancer should be determined. An increased risk of malignancy, particularly non-melanoma skin cancer and non-Hodgkin lymphoma, has been reported in a uveitic population treated with immunomodulatory therapy^(37)^. However, the recently communicated results of a multicenter study indicated no increased risk of overall or cancer-related mortality among patients with uveitis who were receiving common immunomodulatory drugs, including TNF-α inhibitors^(38)^.

Specific vision-threatening conditions, such as VKH syndrome, sympathetic ophthalmia, and Behçet’s disease, allow for the early introduction of immunomodulatory therapy. Other common indications for early use of immunomodulatory drugs include serpiginous choroiditis, birdshot retinochoroidopathy, multifocal choroiditis, and juvenile idiopathic arthritis (JIA)-associated uveitis^(3)^.

When an immunomodulatory drug is not effective in suppressing inflammation, the dose may be increased to the maximum tolerated, or the drug may be combined with or replaced by another drug. The frequent combinations are antimetabolites with T-cell signaling inhibitors or biologic agents^(34,39)^. Simultaneous use of two immunomodulatory drugs from the same category is not recommended because of the increased risk of side effects. Intraocular inflammation should be inactive for at least 2 years before tapering and withdrawing the immunomodulatory therapy^(3)^. The conventional and biologic immunomodulatory drugs used to treat uveitis are presented in tables 4 and 5, respectively.

**Table 4:** Conventional immunomodulatory therapy dosing and side effects

Class	Drug	Dosing	Side effects
Antimetabolites	Methotrexate	Initial dose of 7.5-15 mg/week; up to 25 mg/week PO/SC	GI symptoms, liver toxicity, pulmonary fibrosis, myelosuppression, infection, and phototoxicity
	Mycophenolate mofetil	1-2 g/day PO in divided doses	GI symptoms, hepatotoxicity, infection, and myelosuppression
	Azathioprine	1-3 mg/kg/day PO	GI symptoms, myelosuppression, infection, alopecia, hepatotoxicity, and pancreatitis
	Leflunomide	20 mg/day PO	GI symptoms, hepatotoxicity, and skin rashes
T-cell inhibitors	Cyclosporine	2-3 mg/kg/day PO in divided doses	Tremor, myelosuppression, nephrotoxicity, GI symptoms, arterial hypertension, hirsutism, female reproductive disorder, and gum hyperplasia
	Tacrolimus	0.1-0.2 mg/kg/day PO in divided doses	Tremor, GI symptoms, arterial hypertension, peripheral edema, infection, and nephrotoxicity
Alkylating agents	Cyclophosphamide	2-3 mg/kg/day PO	GI symptoms, myelosuppression, hemorrhagic cystitis, bladder carcinoma, infection, and infertility
	Chlorambucil	0.1-0.2 mg/kg/day PO	GI symptoms, myelosuppression, Stevens-Johnson syndrome, secondary malignancies, infection, and infertility
Other	Sulfasalazine	2g/day PO in divided doses	GI symptoms, blood dyscrasias, and skin rashes

PO= oral; SC= subcutaneous; GI= gastrointestinal.

**Table 5:** Biologic response modifiers, composition, and side effects

Class	Drug	Composition	Side effects	
Anti-TNFα	Infliximab	Chimeric monoclonal Ab	Skin rash, lupus-like illness, vascular thrombosis, congestive heart failure, demyelinating disorders, infections, tuberculosis reactivation, and non-melanoma skin cancers	
	Adalimumab	Human monoclonal Ab	Anaphylaxis, liver toxicity, lupus-like disease, demyelinating disorders, infections, and tuberculosis reactivation	
	Golimumab	Human monoclonal Ab	Skin rash, infections, paresthesia, and hepatotoxicity	
	Certolizumab	Pegylated humanized monoclonal Ab	Skin rash, Stevens-Johnson syndrome, lupus-like disease, infections, and malignancies	
Anti-IL-1β	Anakinra	Recombinant receptor antagonist		GI symptoms, hepatotoxicity, and infections
	Canakinumab	Human monoclonal Ab		GI symptoms, hepatotoxicity, nephrotoxicity, hypersensitivity reactions, and infections
Anti-IL-6	Tocilizumab	Humanized monoclonal Ab (receptor targeted)		GI symptoms, infections, hepatotoxicity, hyperlipidemia, neutropenia, thrombocytopenia, pancreatitis, and anaphylaxis
	Sarilumab	Human monoclonal Ab (receptor targeted)		Infections, myelosuppression, and hepatotoxicity
Anti-IL-17A	Secukinumab	Human monoclonal Ab		GI symptoms, infectious, and urticaria
Anti-CD20	Rituximab	Chimeric monoclonal Ab		Infusion reactions, infections, and progressive multifocal leukoencephalopathy
Interferons	IFN α-2a	Recombinant human protein		GI symptoms, hair loss, thyroid, infections, dysfunction,
	IFN α-2b	Recombinant human protein		depression, leukopenia, thrombocytopenia, and hepatotoxicity
	IFN β	Recombinant human protein		
Other	Intravenous immunoglobulin	Human polyclonal IgG		Eczema, hyperthermia, systemic hypertension, and thrombosis
	Abatacept	Fusion protein		

Varying dosing schedules are used and continue to be adjusted according experience in their use for the treatment of uveitis increases.

TNF= tumor necrosis factor; IL= interleukin; IFN= interferon; Ab= antibody; IV= intravenous; SC= subcutaneous; GI= gastrointestinal.

### Conventional immunomodulatory drugs

#### Antimetabolites

Antimetabolites act by interfering with DNA synthesis and consequently impairing T- and B-cell development^(4)^. The commonly used drugs in this category are methotrexate, mycophenolate mofetil, and azathioprine. In comparison to methotrexate, leflunomide is less frequently used in the treatment of uveitis and has been associated with more recurrences in patients with JIA- associated uveitis^(40)^. Gastrointestinal side effects are the most frequent complaints. Serious complications include hepatotoxicity, pancreatitis, pulmonary fibrosis, myelosuppression, infection, and phototoxicity^(41-43)^.

**Methotrexate** inhibits dihydrofolic acid reductase, interfering with folic acid metabolism and DNA synthesis^(4)^. It is effective for the treatment of a spectrum of noninfectious uveitis subtypes^(41,42)^. A study that compared retention time across conventional systemic immunomodulatory drugs in patients with inflammatory eye disease suggested that methotrexate provided superior effectiveness and tolerability^(44)^. In the Systemic Immunosuppressive Therapy for Eye Disease (SITE) Cohort Study, complete control of uveitis was reported in approximately two-thirds of patients treated with methotrexate and ≤10 mg daily of prednisone within 1 year of systemic treatment^(41)^. Myelosuppression side effects are limited by folic acid supplementation.

**Mycophenolate mofetil** is a prodrug of mycophenolic acid that suppresses guanosine nucleotide synthesis^(4)^. The effectiveness of this drug has been shown in many forms of uveitis^(45)^. The SITE Cohort Study showed that mycophenolate mofetil was effective in controlling inflammation in approximately three-quarters of patients with noninfectious uveitis at 12 months of treatment, although most patients still required corticosteroid treatment^(45)^. Gangaputra et al.^(42)^ suggested that mycophenolate mofetil might be faster than me thotrexate in achieving corticosteroid-sparing success.

**Azathioprine** is a prodrug of 6-mercaptopurine, an analogue of the nucleoside purine^(4)^. Thiopurine S-methyltransferase is an enzyme involved in azathioprine metabolism. Genetic mutation(s) may result in a deficiency of this enzyme that places the patient at risk of side effects due to increased serum levels of azathioprine^(46)^. Sustained control of uveitis was achieved within 12 months in approximately 60% of the patients in the SITE Cohort Study who received azathioprine for uveitis, with a relatively high effectiveness in patients with intermediate uveitis (90%)^(43)^.

#### T-cell signaling inhibitors

Cyclosporine and tacrolimus suppress calcineurin^(4)^, and sirolimus interferes with the mammalian target of rapamycin receptor to downregulate T-cell activity and suppress the expressions of inflammatory cytokines^(4,32)^. Complications include myelosuppression, renal toxicity, gastrointestinal symptoms, systemic hypertension, hirsutism, and gum hyperplasia.

**Cyclosporine** has a history of use in the treatment of Behçet’s disease but is also used for many other forms of noninfectious uveitis^(47)^. More than 50% of patients may achieve inflammation control within the first year, and approximately 10% of patients discontinue treatment owing to toxicity^(47)^.

**Tacrolimus** shares a similar efficacy to cyclosporine (68%) and may have a lower incidence rate of side effects when used to manage noninfectious intermediate and posterior uveitis^(48)^.

#### Alkylating agents

Alkylating agents inhibit DNA replication, limiting the activity of T- and B-cells^(4)^. Cyclophosphamide and chlorambucil are not commonly used, being mostly reserved for cases refractory to antimetabolites and T-cell signaling inhibitors, and recently, to biologic response modifiers because of its serious risks, including infertility and malignancy. Alkylating agents also carry a risk of myelotoxicity and infections.

**Cyclophosphamide** metabolites alkylate the DNA nucleotide guanine, inhibiting DNA replication^(4)^. More than 60% of patients treated with cyclophosphamide achieved remission of noninfectious uveitis within 1 year, but the drug was discontinued in around onethird of the patients as a consequence of adverse side effects^(49)^. One product of cyclophosphamide metabolism is acrolein, a carcinogenic substance excreted in urine; therefore, this treatment carries the risks of hemorrhagic cystitis and bladder carcinoma^(4)^.

**Chlorambucil** produces DNA crosslinking that inhibits cellular replication and induces apoptosis^(4)^. It is usually prescribed for a short period owing to malignancy risk^(50)^. However, results from the largest cohort of patients with uveitis who underwent high-dose chlorambucil therapy during a short-term period showed no malignancies after almost 9 years of mean follow-up^(50,51)^.

#### Others

**Sulfasalazine**, a conjugated molecule of sulfapyridine and 5-aminosalicylate, inhibits prostaglandin synthesis^(52)^. It was evaluated in a small cohort of patients with ankylosing spondylitis-associated anterior uveitis, and the results indicated fewer uveitis recurrences after sulfasalazine therapy, although the grading system used to categorize uveitis activity is no longer used^(52)^.

### Biologic response modifiers

#### Antitumor necrosis factor alpha

Tumor necrosis factor alpha is a cytokine produced by multiple leukocyte subsets and other cell populations that activates several inflammatory pathways^(4)^. Multiple biological response modifiers target TNF-α, but most experiences in the treatment of uveitis have been with infliximab and adalimumab. Adverse reactions to these drugs include skin rash, lupus-like illness, liver toxicity, systemic vascular thrombosis, congestive heart failure, demyelinating disorders, anaphylaxis, and malignancies^(53,54)^. However, data from large numbers of patients with inflammatory bowel disease suggest no increase in the incidence rates of malignancy with anti-TNF-α drugs^(55)^. This issue continues to be investigated in relation to uveitis.

**Infliximab** is a chimeric monoclonal antibody that inhibits TNF-α and is administered intravenously. It has been extensively useed in the treatment of Behçet’s disease and is also effective for the treatment of VKH syndrome, ocular sarcoidosis, HLA-B27-associated uveitis, and retinal vasculitis, with approximately 80% of patients with noninfectious uveitis achieving clinical remission^(53)^. Infliximab has also been successfully used as a “rescue” therapy after failure of other agents, including adalimumab^(56)^.

**Adalimumab** is a human monoclonal anti-TNF-α antibody that is delivered subcutaneously. It has similar efficacy with infliximab in the treatment noninfectious uveitis, with some evidence favoring its use over infliximab in JIA-associated uveitis^(57,58)^. Adalimumab has been approved for the treatment of noninfectious intermediate and posterior uveitis and panuveitis by the United States Food and Drug Administration. Two phase III, randomized, double-masked, placebo-controlled clinical trials (VISUAL I and VISUAL II) demonstrated its effectiveness in delaying time to treatment failure in patients with active and inactive diseases^(59,60)^. Its effectiveness was also demonstrated in SYCAMORE, a randomized, placebo-controlled trial for children with JIA and active uveitis who received methotrexate with placebo or adalimumab^(39)^. Long-term therapy may be necessary for sustained remission, as a 5-year followup report showed that 92% of patients had to resume adalimumab treatment after its withdrawal^(61)^. VISUAL III, a study including some patients from the VISUAL I and II cohorts, demonstrated that among patients with active uveitis, 60% attained ocular inflammation control and 66% discontinued corticosteroid therapy. Threequarters of patients with inactive uveitis at study entry remained in remission at 78 weeks, with 93% of the patients being corticosteroid-free^(62)^.

Adalimumab and infliximab have similar long-term retention rates, which means that infliximab does not show a larger discontinuation proportion than adalimumab^(63)^. However, infliximab requires more clinic visits for intravenous administration, which may be problematic for patients to accommodate in their daily routine. Antidrug antibodies may arise in response to infliximab or adalimumab therapy, which may reduce their effectiveness. Methotrexate or azathioprine administered with anti-TNF-α drugs may decrease the production of antidrug antibodies and improve treatment efficacy^(64)^.

**Golimumab** is a human monoclonal anti-TNF-α antibody, first approved for the treatment of ankylosing spondylitis, rheumatoid arthritis, and psoriatic arthritis^(65-68)^. Successful control of intraocular inflammation has been reported not only in HLA-B27 and JIA-associated uveitis but also in Behçet’s disease and other types of recalcitrant noninfectious uveitis.

**Certolizumab** is a PEGylated, humanized anti-TNF-α monoclonal antigen-binding antibody fragment^(69)^. In patients with spondyloarthritis, it was effective in decrea sing the incidence of uveitis flares in a doublemasked, placebo-controlled study^(70)^. A retrospective survey of patients with noninfectious uveitis found reduced frequency of ocular flares and long-term visual function retention rates, with no side effects, after the administration of certolizumab, including cases of antiTNF-α therapy failure^(70)^.

**Etanercept** is a TNF-α receptor-based drug that is used in the treatment of rheumatoid arthritis and spondyloarthropathies. However, it shows little effectiveness in controlling intraocular inflammation and has been associated with paradoxical uveitis; therefore, its use is not recommended for uveitis therapy^(71,72)^.

*Anti-IL-1*β

**Canakinumab** is a human anti-IL-1β monoclonal antibody, whereas **anakinra** is a recombinant humanized IL-1 receptor antagonist. Both drugs limit the effect of IL-1β, a potent inflammatory cytokine. The effectiveness of both drugs was described in the treatment of Behçet’s disease, with gastrointestinal symptoms, infections, and anaphylaxis as reported side effects^(73,74)^.

**Gevokizumab** is a recombinant humanized antiIL-1β antibody, which despite having shown promising results in severe Behçet’s uveitis in a phase II study, did not progress to clinical application after its effectiveness was not confirmed in randomized, double-masked, placebo-controlled phase III trials ^(75,76)^. The drug did not decrease the number of uveitis flares in Behçet’s disease, although results suggested that vision was preserved and cystoid macular edema was reduced in the first 6 months of treatment.

#### Anti-IL-6

**Tocilizumab** is a humanized monoclonal antibody against the receptor of IL-6, a cytokine with a broad range of inflammatory activities^(77)^. It is administered by intravenous infusion or subcutaneously. Studies of tocilizumab conducted in patients with noninfectious uveitis and macular edema refractory to conventional immunomodulatory drugs, anti-TNF-α agents, and interferons have shown favorable outcomes in Behçet’s disease, birdshot retinochoroidopathy, JIA-associated uveitis, sympathetic ophthalmia, ocular sarcoidosis, and idiopathic panuveitis. However, the phase II APTITUDE trial, which assessed tocilizumab in 21 patients with JIA-associated uveitis refractory to antiTNF-α treatment, did not meet the efficacy end point required for phase III trials, despite demonstrating a good safety profile^(78)^. Reported side effects include gastrointestinal symptoms, upper respiratory tract infections, hepatotoxicity, hyperlipidemia, neutropenia, and thrombocytopenia^(77,79-81)^.

**Sarilumab** is a human anti-IL-6 receptor monoclonal antibody that was found to be well tolerated and effective for improving vision, reducing inflammation, and decreasing corticosteroid use in adults with noninfectious uveitis when compared with placebo^(82)^.

#### Anti-IL-17

**Secukinumab** is a human monoclonal anti-IL-17A antibody, available in both intravenous and subcutaneous preparations^(83)^. Three phase III clinical trials of subcutaneous administered secukinumab in patients with noninfectious uveitis failed to meet the primary efficacy end points^(83)^. By contrast, the results of a subsequent phase II clinical trial, which involved intravenous and subcutaneous drug administration, suggested that intravenous secukinumab had therapeutic benefits in patients with uveitis, reducing inflammation in up to 73% and achieving remission in around a third of all cases, with a good safety profile^(84)^.

#### Anti-CD20

**Rituximab** is a chimeric monoclonal anti-CD20 antibody that targets B-cells and has been administered intravenously for noninfectious uveitis^(85,86)^. It also has been successfully used in the management of refractory cases of orbital inflammation and scleritis^(87,88)^, and intravitreally for ocular involvement in primary central nervous system lymphoma^(30)^. Rituximab is generally well tolerated, with infusion reactions being the most common adverse event. Infections are surprisingly uncommon. Progressive multifocal leukoencephalopathy is a rare complication.

#### Interferons

After binding the cell membrane receptor, interferons initiate signal transduction cascades that stimulate the production of proteins with antiviral and immunomodulatory properties^(4)^. Flu-like symptoms are the commonly reported side effects of interferon, but severe adverse events are rare. Other reported complications are gastrointestinal disturbance, hair loss, dysthyroidism, depression, leukopenia, thrombocytopenia, and elevated liver enzyme levels^(4)^.

**Interferon-**α **2a** is a recombinant interferon used in various regimens. Effective control of inflammation in >80% of patients with persistent Behçet’s uveitis has been shown^(89)^. A retrospective study found a 62.5% response rate to interferon-α 2a used for recalcitrant macular edema^(90)^. However, a randomized controlled intention-to-treat trial that evaluated treatment using interferon-α 2a with corticosteroids compared with no treatment found no significant difference in the reduction of macular edema in chronic noninfectious uveitis at 4 months^(91)^.

The composition of **interferon-**α **2b** differs from that of interferon α-2a by one amino acid. In one small case series, 4 patients with persistent Behçet’s uveitis were treated successfully with PEGylated interferon-α 2b^(92)^. In another series of 4 patients, a radical improvement in cystoid macular edema secondary to uveitis was observed^(93)^.

**Interferon-**β is approved for the treatment of multiple sclerosis and delivered by subcutaneous or intramuscular injection^(94)^. A few small cohort studies have evaluated the effects of interferon-β on uveitis. In patients with multiple sclerosis and uveitis who were receiving interferon-β, improvement in vision and reduction in macular edema were reported^(94)^. However, more severe uveitis was also observed with interferon-β treatment when compared with no treatment^(95)^. A prospective randomized trial with 19 patients that compared interferon-β and methotrexate for the treatment of macular edema in intermediate uveitis identified interferon-β as the superior treatment after 3 months^(96)^.

#### Intravenous immunoglobulin

**Intravenous immunoglobulin** represents human immunoglobulin (Ig) G acquired through the donation of plasma by healthy donors. It has immunomodulatory functions that affect T- and B-cell responses, and is applied in the treatment of immunodeficiencies and systemic inflammatory diseases^(97)^. In uveitis, intravenous immunoglobulin was found to be effective in controlling inflammation and improving vision in patients with Behçet’s disease, birdshot retinochoroidopathy, cancerassociated retinopathy, and other forms of refractory noninfectious uveitis^(98-100)^. Regimens of infusions are repeated every 1-2 months at considerable costs. Possible side effects are eczema, hyperthermia, systemic hypertension, and thrombosis.

#### T-cell activation blocker

**Abatacept** is a selective co-stimulation modulator, created from a portion of human IgG1 fused to the portion of cytotoxic T-lymphocyte-associated protein (CTLA)-4 that binds CD80 or CD86 on antigen-presenting cells, and hence is capable of preventing the co-stimulatory signal required for T-cell activity^(101)^. It is available as subcutaneous or intravenous injections. Despite the early promising data for the treatment of refractory uveitis^(93)^, a retrospective study of 21 patients with JIA and refractory uveitis who were receiving abatacept reported remission in just 11 patients, with later recurrence in 8^(101,102)^.

#### Janus kinases inhibitors

Janus kinases (JAKs) are enzymes that participate in intracellular signaling activated by inflammatory cytokines^(103)^. At least four JAKs exist, namely JAK1, JAK2, JAK3, and TYK2. Tofacitinib and baricitinib are JAK inhibitors that are already being quite widely used in the treatment of rheumatoid arthritis. In one small series of 4 patients with severe JIA-associated uveitis, intraocular inflammation improved in all following initiation of baricitinib or tofacitinib therapy, although joint inflammation improved in just one patient^(104)^. Clinical trials of several JAK inhibitors for noninfectious uveitis are currently in progress.

Despite the vast arsenal of drugs currently available for the treatment of noninfectious uveitis, an ideal therapy that induces sustained remission even after withdrawal is still lacking. The development of biologic response modifiers established a new era in the treatment of uveitis, with a large number of agents on the market and continuous release of more drugs. These drugs are aimed at specifically modulating inflammation to minimize the adverse reactions that may be encountered when using conventional immunomodulatory agents or corticosteroids. As research advances and new discoveries are made on the complex immunologic mechanisms of intraocular inflammation, additional targets are likely to emerge for future biologic therapies of noninfectious uveitis.
